# Associative Memory Extinction Is Accompanied by Decayed Plasticity at Motor Cortical Neurons and Persistent Plasticity at Sensory Cortical Neurons

**DOI:** 10.3389/fncel.2017.00168

**Published:** 2017-06-14

**Authors:** Rui Guo, Rongjing Ge, Shidi Zhao, Yulong Liu, Xin Zhao, Li Huang, Sodong Guan, Wei Lu, Shan Cui, Shirlene Wang, Jin-Hui Wang

**Affiliations:** ^1^Department of Pathophysiology, Bengbu Medical CollegeAnhui, China; ^2^School of Pharmacy, Qingdao UniversityQingdao, China; ^3^Brain and Cognitive Sciences, Institute of Biophysics, Chinese Academy of SciencesBeijing, China; ^4^Department of Psychiatry and Behavioral Sciences, Feinberg School of Medicine, Northwestern UniversityChicago, IL, United States; ^5^Department of Biology, University of Chinese Academy of SciencesBeijing, China

**Keywords:** learning, memory, glutamate, GABA, neuron, synapse, barrel cortex and homeostasis

## Abstract

Associative memory is essential for cognition, in which associative memory cells and their plasticity presumably play important roles. The mechanism underlying associative memory extinction vs. maintenance remains unclear, which we have studied in a mouse model of cross-modal associative learning. Paired whisker and olfaction stimulations lead to a full establishment of odorant-induced whisker motion in training day 10, which almost disappears if paired stimulations are not given in a week, and then recovers after paired stimulation for an additional day. In mice that show associative memory, extinction and recovery, we have analyzed the dynamical plasticity of glutamatergic neurons in layers II–III of the barrel cortex and layers IV–V of the motor cortex. Compared with control mice, the rate of evoked spikes as well as the amplitude and frequency of excitatory postsynaptic currents increase, whereas the amplitude and frequency of inhibitory postsynaptic currents (IPSC) decrease at training day 10 in associative memory mice. Without paired training for a week, these plastic changes are persistent in the barrel cortex and decayed in the motor cortex. If paired training is given for an additional day to revoke associative memory, neuronal plasticity recovers in the motor cortex. Our study indicates persistent neuronal plasticity in the barrel cortex for cross-modal memory maintenance as well as the dynamical change of neuronal plasticity in the motor cortex for memory retrieval and extinction. In other words, the sensory cortices are essential for long-term memory while the behavior-related cortices with the inability of memory retrieval are correlated to memory extinction.

## Introduction

Associative learning is a common approach for information acquisition and associative memory is essential to logical reasoning and associative thinking (Wasserman and Miller, 1997; Suzuki, 2008; Wang and Cui, 2017). In terms of cellular mechanisms underlying associative learning and memory, associative memory cells are recruited in co-activated sensory cortices (Wang et al., 2015; Gao et al., 2016; Vincis and Fontanini, 2016; Yan et al., 2016) and their downstream brain regions (Naya et al., 2003; Takehara-Nishiuchi and McNaughton, 2008; Viskontas, 2008; Cai et al., 2016), as well as use-dependent neural plasticity occurs during associative memory (Honey and Good, 2000; Blair et al., 2001; Christian and Thompson, 2003; Jones et al., 2003; Silva, 2003; Zhang et al., 2004; Dityatev and Bolshakov, 2005; Fanselow and Poulos, 2005; Weeks et al., 2007; Frey and Frey, 2008; Nikitin et al., 2008; Rosselet et al., 2011; Gao et al., 2016; Yan et al., 2016). On the other hand, memory extinction remains to be mechanistically elucidated, since it is necessary to know why memory losses occur during the mental retardation of neurological and psychological diseases, and how fear memory that leads to anxiety and major depression can be removed from the brains (Myers and Davis, 2002; Orsini and Maren, 2012; Baldi and Bucherelli, 2015; Giustino et al., 2016; Knox, 2016). If information storage is based on memory cells and their plasticity, the decreases in the level of neural plasticity and the number of memory cells may be associated with memory retrieval inability and even extinction.

In the evaluation of memory extinction, the inability of information retrieval and recall through behavioral presentation is considered to be memory loss (Cammarota et al., 2005; Almeida-Corrêa and Amaral, 2014). Although the stored information cannot be recalled automatically and intentionally sometimes, its recall can be induced by the cues similar to or equal to the identities of primarily learned objects or events, indicating the information retention in the brain. This phenomenon suggests that the persistent storage of the learned information in certain brain areas and the attenuated ability to represent the stored information in behavior-related brain areas may be involved in memory extinction. It has been suggested that information retrievals triggered by the cues and presented by the behaviors are fulfilled by the neuronal circuits from sensory cortices to behavior-guide cortices through their relayed brain regions (Wang et al., 2015). This suggestion is granted by the facts that the stimulations to any of these areas can trigger memory retrievals (Ehrlich et al., 2009; Pape and Pare, 2010; Liu et al., 2012; Li et al., 2013; Xu and Südhof, 2013; Otis et al., 2017; Yokose et al., 2017) as well as the responses to associated signals can be recorded in sensory cortices (Wang et al., 2015; Vincis and Fontanini, 2016; Yan et al., 2016) and their downstream brain regions (Naya et al., 2003; Takehara-Nishiuchi and McNaughton, 2008; Viskontas, 2008; Cai et al., 2016). In other words, the sensory cortices are still primary locations for the signal storage and retrieval initiation (Wang et al., 2015; Gao et al., 2016). If this is a case, we should see the persistence of neuronal plasticity in the sensory cortices and the decay of neuronal plasticity in behavior-control cortices during memory extinction, as well as the recovery of neuronal plasticity in behavior-control cortices after memory restoration.

To approach these questions above, we aimed to investigate cellular mechanisms underlying memory formation and retrieval inability in a mouse model of associative learning (Wang et al., 2015) by comparing neuronal plasticity at the sensory and motor cortices during the periods of associative memory establishment, extinction and reestablishment. Neuronal plasticity was analyzed based on synaptic transmission and spiking capability by whole-cell recordings at glutamatergic neurons in the barrel and motor cortices, which were genetically labeled by yellow fluorescent protein (YFP) for the identification of these neurons (Feng et al., 2000) under the fluorescent microscope.

Memory extinction stands for the loss of the stored information. Memory retrieval inability is termed as that the stored information is unable to be retrieved.

## Materials and Methods

All experimental protocols were performed in accordance with the guidelines by the Administration Office of Laboratory Animals at Beijing China. All experiment protocols were approved by the Institutional Animal Care Unit Committee in the Administration Office of Laboratory Animals at Beijing China (B10831).

### Mouse Model of Associative Memory

To analyze cell-specific mechanism for associative memory we used C57 Thy1-YFP mice (Feng et al., 2000; Zhang et al., 2013), whose glutamatergic neurons were genetically labeled by YFP.

Two groups of mice were trained at postnatal days 20 by the simultaneous pairing of mechanical whisker stimuli (WS) with odor stimuli (OS, butyl acetate toward the noses) and the unpairing of these stimuli (control), respectively (Wang et al., 2015; Gao et al., 2016; Yan et al., 2016). The stimulations for paired WS/OS and unpaired WS/OS mice were given by the multiple-sensory modal stimulator (MSMS, ZL201410499466), in which the intensities, time, frequency and intervals of OS and WS were precisely and consistently set. In unpairing group, the interval between WS and OS was randomly about 2–5 min. The OS intensity was sufficient to induce the response of olfactory bulb neurons seen by two-photon Ca^2+^ imaging, and the WS intensity was sufficient to evoke whisker fluctuation after WS ended. Each of these mice was trained 20 s in each time, five times per day with intervals of 2 h for consecutively 10 days. It is noteworthy that the intensities, time, frequency and total number of the WS and OS are same for paired and unpaired groups. During the training, each mouse was placed in a home-made cage. Care was taken to avoid stressful experimental condition and circadian disturbance to the mice that showed normal whisking and symmetric whiskers (Wang et al., 2015; Gao et al., 2016; Yan et al., 2016). Long whiskers (such as arcs 1–2) on the same side and rows were assigned for mechanical stimuli and for the observation of their responses to the odor-test. This selection was based on studies in cross-modal plasticity (Ni et al., 2010; Ye et al., 2012). We did not trim short whiskers since whisker trimming elevated the excitability of the barrel cortex (Zhang et al., 2013).

Whisker motion tracks were monitored by a digital video camera (240 Hz) and were quantified in whisker retraction angle and whisking frequency (MB-Ruler, version 5.0 by Markus Bader, MB-Softwaresolution, Germany). Whisking angles were measured as angles lined from the original position to whisker retraction. Whisking frequency was the times of whisker fluctuation per second (Hz). The response of mouse whiskers to the odor-test (butyl acetate, 20 s) was measured before the training and at the end of each training day to quantify the onset time and levels of conditioned reflex (CR). CR-formation was defined to meet the following criteria. The patterns of odorant-induced whisker motion were similar to those of WS-induced whisker motion. Whisking frequency and angles significantly increased, compared to those before the training. The approaches for statistical analyses are given in the section of *statistical analysis*. As this type of whisker motion induced by the odorant was originally induced by WS, the odor signal initiated a recall of the whisker signal and then led to whisker motion (Wang et al., 2015; Gao et al., 2016; Yan et al., 2016).

**Figure 1:**
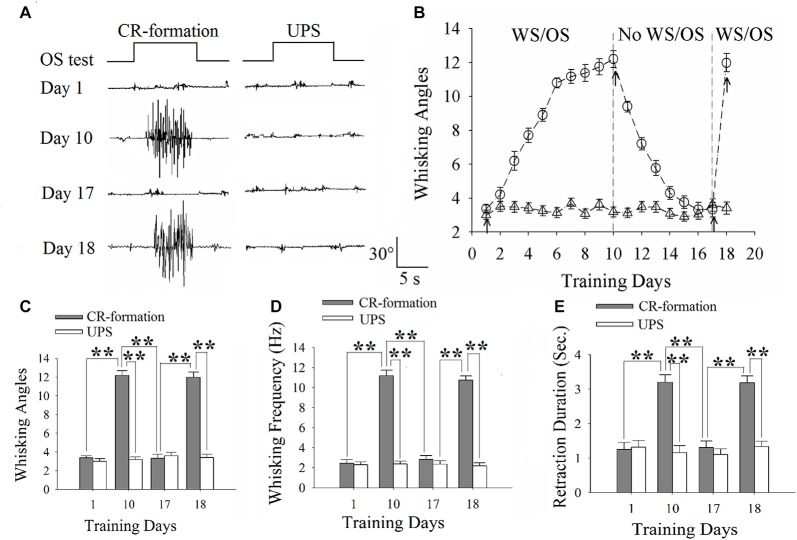
The establishment, extinction and reestablishment of odorant-induced whisker motion in mice. **(A)** Presents whisker motions in response to the odor-test (black traces on top) in conditioned reflex (CR)-formation mice and unpaired stimulus (UPS) mice at the days 1, 10, 17 and 18. The paired or unpaired trainings were given from day 1 to day 10 as well as at day 18, however, the non-training was between day 11 and 16. Calibration bars are 30° and 5 s. **(B)** illustrates the angles of whisker fluctuation in response to the odor-test during different training days in CR-formation mice (round symbols) and UPS mice (triangles), indicating odorant-induced whisker motion in terms of establishment, decay and reestablishment. **(C)** illustrates the comparisons of whisking angles in response to the odor-test in CR-formation mice (filled bars) and UPG mice (hollow bars) at training days 1, 10, 17 and 18. **(D)** shows the comparisons of whisking frequencies in response to the odor-test in CR-formation mice (filled bars) and UPG mice (hollow bars) at training days 1, 10, 17 and 18. **(E)** illustrates the comparisons of whisker retraction duration in response to the odor-test in CR-formation mice (filled bars) and UPG mice (hollow bars) at the training days 1, 10, 17 and 18. Two asterisks show *p* < 0.01 (Statistical significance was determined using two-way analysis of variance (ANOVA) with a Bonferroni correction for multiple comparisons).

The group of WS/OS-paired mice showing odorant-induced whisker motion was further divided into three subgroups for behavioral tests to show associative memory establishment, extinction and reestablishment as well as for electrophysiological study in the barrel and motor cortices to show dynamic changes in neuronal plasticity. That is, the mice show the establishment of odorant-induced whisker motion trained by WS/OS-pairing for 10 days, the mice show the decay of associative memory (odorant-induced whisker motion trained by the WS/OS pairing for 10 days) without further training in a subsequent week, and the mice show the reestablishment of associative memory by WS/OS-paired training for an additional day after the decay of odorant-induced whisker motion, as illustrated in Figure 1. These three groups of the mice were used for the studies of neuronal activities by electrophysiology.

### Brain Slices and Neurons

Cortical slices (400 μm) were prepared from the mice of CR-formation, CR-extinction and CR-recovery within 24 h after the training was ended. They were anesthetized by inhaling isoflurane and decapitated by the guillotine. The slices were cut by Vibratome in the oxygenated (95%O_2_/5%CO_2_) artificial cerebrospinal fluid (ACSF), in which the chemical concentrations (mM) were 124 NaCl, 3 KCl, 1.2 NaH_2_PO_4_, 26 NaHCO_3_, 0.5 CaCl_2_, 4 MgSO_4_, 10 dextrose and 5 HEPES, pH 7.35 at 4°C. The slices were held in the oxygenated ACSF (124 NaCl, 3 KCl, 1.2 NaH_2_PO_4_, 26 NaHCO_3_, 2.4 CaCl_2_, 1.3 MgSO_4_, 10 dextrose and 5 HEPES, pH 7.35) at 25°C for 2 h. The slices were transferred to submersion chamber (Warner RC-26G) that was perfused with the oxygenated ACSF at 31°C for whole-cell recording (Wang and Kelly, 2001).

Electrophysiological recordings on YFP-labeled glutamatergic neurons in layers II-III of the barrel cortices as well as layers IV–V of the motor cortices were conducted under a DIC-fluorescent microscope (Nikon FN-E600, Japan). The wavelength at 575 nm excited YFP. These glutamatergic neurons showed pyramidal shape and regular spikes with adaptations of spike amplitudes and frequencies (DeFelipe et al., 2013; Lu et al., 2014; Xu et al., 2016). The cerebral slices were the coronal sections including the barrels correspondent to the projection from long whiskers that were stimulated in pairing WS and OS training.

### Whole-Cell Recording

Cortical neurons were recorded by MultiClamp-700B amplifier in voltage-clamp for their synaptic activities. The electrical signals were inputted into pClamp-10 (Axon Instrument Inc., CA, USA) for data acquisitions and analyses. The output bandwidth in this amplifier was 3 kHz. The pipette solution for studying excitatory synapses included (mM) 150 K-gluconate, 5 NaCl, 5 HEPES, 0.4 EGTA, 4 Mg-ATP, 0.5 Tris-GTP and 5 phosphocreatine (pH 7.35; (Ge et al., 2011, 2014)). The pipette solution for studying the inhibitory synapses contained (mM) 130 K-gluconate, 20 KCl, 5 NaCl, 5 HEPES, 0.5 EGTA, 4 Mg-ATP, 0.5 Tris–GTP and 5 phosphocreatine (Zhang et al., 2012). These pipette solutions were freshly made and filtered (0.1 μm), in which their osmolarity was 295–305 mOsmol and pipette resistance was 5–6 MΩ.

The functions of the glutamatergic neurons were assessed based on their active intrinsic property, excitatory synaptic transmission and inhibitory synaptic transmission (Chen et al., 2008; Wang et al., 2008). The excitatory synaptic transmission was evaluated by recording spontaneous excitatory postsynaptic currents (sEPSCs) under the voltage-clamp on these glutamatergic neurons in presence of 10 μM bicuculline in ACSF to block ionotropic GABA receptors (Wang, 2003). Ten micro molar CNQX and 40 μM DAP-5 were added into ACSF perfused onto the slices at the end of experiments to examine whether the synaptic responses were mediated by GluRs, which blocked EPSCs in our experiments. Series and input resistances for all neurons were monitored by injecting hyperpolarization pulses (5 mV/50 ms), and calculated by voltage pulses vs. instantaneous and steady-state currents.

Inhibitory synaptic transmission was evaluated by recording spontaneous inhibitory postsynaptic currents (sIPSCs) under the voltage-clamp on glutamatergic neurons in the presence of 10 μM 6-Cyano-7-nitroquinoxaline-2,3-(1H,4H)-dione (CNQX) and 40 μM D-amino-5-phosphonovanolenic acid (D-AP5) in ACSF to block ionotropic glutamate receptors (Wei et al., 2004; Ma et al., 2016a). Ten micro molar bicuculline was washed onto the slices at the end of experiments to examine whether synaptic responses were mediated by GABA_A_R, which blocked sIPSCs in our experiments. Series and input resistances in all of the neurons were monitored by injecting hyperpolarization pulses (5 mV/50 ms), and calculated by voltage pulses vs. instantaneous and steady-state currents. The pipette solution with the high concentration of chloride ions makes the reversal potential to be −42 mV. sIPSCs will be inward when the membrane holding potential at −65 (Wei et al., 2004; Wang G. Y. et al., 2016; Xu et al., 2016).

Action potentials at these cortical neurons were induced by injecting depolarization pulses, whose intensity and duration were altered based on the aim of the experiments. The ability to convert excitatory inputs into digital spikes was evaluated by input-output (spikes per second vs. normalized stimuli) when various stimuli were given (Chen et al., 2006a,b, 2008; Wang et al., 2008), in which stimulus intensities were step-increasing by 10% normalized stimulations. As the excitability of different neurons was variable, step-increased depolarization pulses were given based on their normalization. The base value of stimulus intensity for this normalization at each neuron was the threshold intensity of depolarization pulse (1000 ms in duration) to evoke a single spike (Chen et al., 2006b). We did not measure the rheobase to show neuronal excitability, since this strength-duration relationship was used to indicate the ability to fire a single spike.

The recordings of spontaneous synaptic currents, instead of the evoked synaptic currents, are based on the following reasons. sEPSC and sIPSC amplitudes represent the responsiveness and the densities of the postsynaptic receptors. The frequencies imply the probability of transmitter release from an axon terminal and the number of the presynaptic axons innervated on the recorded neuron (Zucker and Regehr, 2002; Stevens, 2004). These parameters can be used to analyze presynaptic and postsynaptic mechanisms underlying neuronal plasticity, whereas the evoked postsynaptic currents cannot separate these mechanisms. We did not add TTX into the ACSF to record miniature postsynaptic currents since we had to record neuronal excitability (Ma et al., 2016b; Xu et al., 2016).

Data were analyzed if the recorded neurons had resting membrane potentials negatively more than −70 mV and action potential amplitudes more than 100 mV. The criteria for the acceptance of each experiment also included less than 5% alternations in the resting membrane potential, spike magnitude, and input resistance throughout each experiment. Input resistance was monitored by measuring cellular responses to hyperpolarization pulse at the same values as the depolarization that evoked action potentials. In order to estimate the effects of associative learning on neuronal spikes and synaptic transmission, we measured the amplitudes and intervals of sEPSC and sIPSC as well as the input-output of spikes vs. stimulations under the conditions of control and associative memory including its establishment, extinction and reestablishment, which were presented as mean ± SE. sEPSC and sIPSC frequencies were calculated by 1/intervals. The comparisons of sEPSCs and sIPSCs among different groups were based on their values at 67% of cumulative probability.

### Statistical Analyses

The paired *t*-test was used in the comparisons of the experimental data before and after associative learning, before and after bicuculine or CNQX/D-AP5 applications, as well as the neuronal responses to whisker stimulus and odorant stimulus in each of the mice. One-way analysis of variance (ANOVA) was applied to make the statistical comparisons in the changes of neuronal activities between control and CR-formation groups.

## Results

### Dynamical Changes in Establishment, Extinction and Reestablishment of Odorant-Induced Whisker Motion

Mice were trained by pairing WS and OS or WS/OS-unpaired stimulation (UPS; Wang et al., 2015; Gao et al., 2016; Yan et al., 2016). In WS/OS-paired mice, their whiskers respond to the odor-test after paired trainings for 10 days, i.e., odorant-induced whisker motion or CR. This CR-formation disappears without WS/OS-paired training in a week, and can be reevoked by the WS/OS-paired training for additional day (Figure 1A). Figure 1B shows statistical data about this odorant-induced whisker motion that is fully establishment at training day 10, decays within 1 week and recovers by WS/OS-pair for an additional day (circle symbols in Figure 1B; *n* = 10), compared with UPS mice (triangles; *n* = 10). Figures 1C–E shows whisking angle, whisking frequency and whisker retraction duration in CR-formation mice (filled bars) and UPS mice (hollow bars) at days 1, 10, 17 and 18. Whisking angles are 3.37 ± 0.22° at day 1, 12.20 ± 0.50° at day 10, 3.32 ± 0.43° at day 17 and 11.98 ± 0.54° at day 18 (Figure 1C). Whisking frequencies are 2.45 ± 0.37 Hz at day 1, 11.18 ± 0.54 Hz at day 10, 2.45 ± 0.37 Hz at day 17 and 10.75 ± 0.4 Hz at day 18 (Figure 1D). Whisker retraction durations are 1.25 ± 0.2 s at day 1, 3.20 ± 0.20 s at day 10, 1.31 ± 0.20 s at day 17 and 3.19 ± 0.2 s at day 18 (Figure 1E; two asterisks denote *p* < 0.01; One-way ANOVA). Therefore, odorant-induced whisker motion shows a full establishment by the WS/OS-pairing for 10 days, the extinction without the WS/OS-pairing for 1 week and the reestablishment by the WS/OS-pairing for an additional day.

In terms of cellular mechanisms underlying the establishment, extinction and reestablishment of cross-modal associative memory in CR-formation mice, we hypothesize that neuronal plasticity in cerebral cortices establishes, decays and reestablishes. As odorant-induced whisker motion is accompanied by the upregulations of glutamatergic neurons and synapses in the barrel cortices (Wang et al., 2015; Gao et al., 2016; Yan et al., 2016), we aim to study whether the upregulation, decay and re-upregulation of neuronal and synaptic activity in the barrel cortex and the motor cortex are parallel to and even correlated to the establishment, extinction and reestablishment of cross-modal associative memory.

The analyses of neural plasticity at glutamatergic neurons included the functional changes in their ability to convert analog synaptic signals into digital spikes as well as their receptions to excitatory and inhibitory synaptic inputs in CR-formation mice and UPS mice. In the coronal directions of brain slices including the barrel cortex (glutamatergic neurons in layers II–III) or the motor cortex (glutamatergic neurons in layers IV–V), sEPSC were recorded by whole-cell voltage-clamp to assess excitatory synaptic transmission. Input-output curves at these neurons were measured under current-clamp to evaluate their ability to convert excitatory inputs into spikes. sIPSCs were recorded to assess inhibitory synaptic function (Zhang et al., 2013; Gao et al., 2016).

### Persistent Maintenance of Spiking Ability at Barrel Cortical Neurons, but Not Motor Cortical Neurons

For studying the roles of glutamatergic neurons at barrel and motor cortices in memory maintenance and extinction, the mice that showed odorant-induced whisker motion were divided into three groups: CR-formation by WS/OS-paired training for 10 days (day 10), CR-formation by WS/OS-paired training for 10 days and then without WS/OS-pairing in a week for CR-extinction (day 17), as well as CR-recovery by WS/OS-paired training for an additional day (day 18). Their functional plasticity was compared with that in UPS mice.

**Figure 2:**
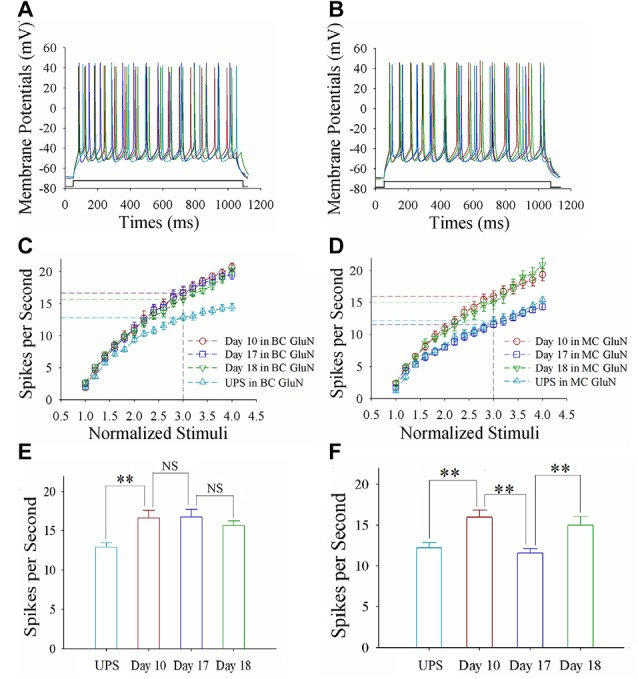
The enhanced ability to encode spikes is maintained at barrel cortical glutamatergic neurons, but not at motor cortical glutamatergic neurons. In the UPS mice and CR-formation mice at training days 10, 17 and 18, the sequential spikes were induced by depolarization pulses under the current-clamp recording on the yellow fluorescent protein (YFP)-labeled glutamatergic neurons in brain slices. **(A)** illustrates the spikes induced by a depolarization pulse in barrel cortical glutamatergic neurons from UPS mouse (cyan trace) and from CR-formation mice at training days 10 (red), 17 (blue) and 18 (green). **(B)** illustrates the spikes induced by a depolarization pulse in motor cortical glutamatergic neurons from UPS mouse (cyan trace) and from CR-formation mice at training days 10 (red), 17 (blue) and 18 (green). **(C)** illustrates spikes per second vs. normalized stimuli in the barrel cortical neurons from UPS mouse (cyan symbols) and from CR-formation mice at training days 10 (red), 17 (blue) and 18 (green). **(D)** shows spikes per second vs. normalized stimuli in the motor cortical neurons from UPS mouse (cyan symbols) and from CR-formation mice at training days 10 (red), 17 (blue) and 18 (green). **(E)** shows statistical comparisons of spikes per second at 3.0 normalized stimuli in the barrel cortical neurons from UPS mouse (cyan bar, 12.92 ± 0.54, *n* = 13) and from CR-formation mice at training days 10 (red, 16.64 ± 0.95, *n* = 11), 17 (blue, 16.75 ± 0.98, *n* = 12) and 18 (green, 15.70 ± 0.56, *n* = 10; from left: *p* = 0.004, *p* = 0.934, *p* = 0.388). **(F)** shows statistical comparisons of spikes per second at 3.0 normalized stimuli in motor cortical neurons from UPS mouse (cyan bar, 12.2 ± 0.61, *n* = 10) and from CR-formation mice at training days 10 (red, 16 ± 0.86, *n* = 12), 17 (blue, 11.58 ± 0.53, *n* = 12) and 18 (green, 15 ± 1.05, *n* = 11; from left: *p* = 0.002, *p* = <0.001, *p* = 0.011). A one-way ANOVA with Bonferroni correction for multiple comparisons was performed to test for significant changes, two asterisks represent *p* < 0.01, NS represents no statistical significance.

Sequential spikes on glutamatergic neurons from barrel and motor cortices were induced by depolarization pulse in CR-formation mice at training days 10, 17 and 18 as well as UPS mice (Figures 2A,B). Figure 2C shows spikes per second vs. normalized stimuli in barrel cortical neurons from CR-formation mice at training day 10 (red symbols; *n* = 11 cells from 6 mice), day 17 (blue; *n* = 12 cells from 6 mice) and day 18 (green; *n* = 10 cells from 7 mice), as well as those from UPS mice (cyan; *n* = 13 cells from 6 mice). Figure 2D shows spikes per second vs. normalized stimuli in motor cortical neurons from CR-formation mice at training day 10 (red symbols, *n* = 12), day 17 (blue, *n* = 12) and day 18 (green, *n* = 11), as well as those from UPS mice (cyan, *n* = 10). Spikes per second at the 3.0 of normalized stimuli in barrel cortical neurons from CR-formation mice are 16.64 ± 0.95 at day 10 (red bar in Figure 2E), 16.75 ± 0.98 at day 17 (blue) and 15.70 ± 0.56 at day 18 (green), compared to 12.92 ± 0.54 at those neurons from UPS mice (cyan). Spikes per second at the 3.0 of normalized stimuli in motor cortical neurons from CR-formation mice are 16 ± 0.86 at day 10 (red bar in Figure 2F), 11.58 ± 0.53 at day 17 (blue) and 15 ± 1.05 at day 18 (green), compared with 12.2 ± 0.61 at those from UPS mice (cyan). These results indicate that the increased spiking ability in barrel cortical glutamatergic neurons is maintained regardless of the extinction of cross-modal associative memory, while spiking ability in motor cortical glutamatergic neurons is decayed in the retrieval inability of associative memory.

### Excitatory Synapse Upregulation and Inhibitory Synapse Downregulation Maintain at Barrel Cortical Neurons

**Figure 3:**
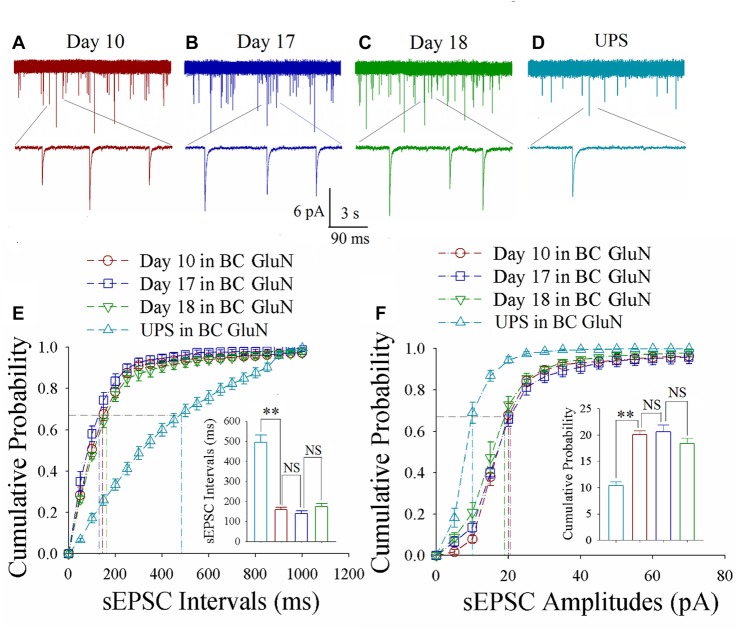
Enhanced excitatory synaptic transmission is maintained at barrel cortical glutamatergic neurons. In UPS mice and CR-formation mice at training days 10, 17 and 18, spontaneous excitatory postsynaptic currents (sEPSCs) were recorded under voltage-clamp recording on YFP-labeled glutamatergic neurons in brain slices. **(A)** shows sEPSCs at barrel cortical glutamatergic neuron from a CR-formation mouse at training day 10. **(B)** illustrates sEPSCs at barrel cortical glutamatergic neuron from a CR-formation mouse at training day 17. **(C)** shows sEPSCs at barrel cortical glutamatergic neuron from a CR-formation mouse at training day 18. **(D)** illustrates sEPSCs at barrel cortical glutamatergic neuron from UPS mouse. **(E)** shows cumulative probability vs. sEPSCs intervals in the barrel cortical neurons from UPS mouse (cyan symbols) and from CR-formation mice at training days 10 (red), 17 (blue) and 18 (green). Insert shows sEPSC intervals at 67% cumulative probability in the barrel cortical neurons from UPS mouse (cyan bar, 494 ± 36 ms, *n* = 12) and from CR-formation mice at training days 10 (red, 158 ± 11 ms, *n* = 11), 17 (blue, 138 ± 14 ms, *n* = 11) and 18 (green, 174 ± 15 ms, *n* = 9; from left: *p* = <0.001, *p* = 0.272, *p* = 0.094). **(F)** illustrates cumulative probability vs. sEPSC amplitudes in barrel cortical neurons from UPS mouse (cyan symbols) and from CR-formation mice at training days 10 (red), 17 (blue) and 18 (green). Insert shows sEPSC amplitudes at 67% cumulative probability in the barrel cortical neurons from UPS mouse (cyan bar, 10.4 ± 0.7 pA, *n* = 12) and from CR-formation mice at training days 10 (red, 20.0 ± 0.80 pA, *n* = 11), 17 (blue, 20.6 ± 1.30 pA, *n* = 11) and 18 (green, 18.30 ± 1.10 pA, *n* = 9; from left: *p* < 0.001, *p* = 0.612, *p* = 0.092). A one-way ANOVA with Bonferroni correction for multiple comparisons was performed to test for significant changes, two asterisks represent *p* < 0.01, NS represents no statistical significance.

As shown in Figures 3A–D, spontaneous excitatory synaptic currents were recorded by whole-cell voltage-clamp on barrel cortical glutamatergic neurons in CR-formation mice at training days 10, 17 and 18 as well as those in UPS mice. Compared with UPS mice (cyan), sEPSC amplitude and frequency appear increased in days 10 (red trace), 17 (blue) and 18 (green). Figure 3E shows cumulative probability vs. sEPSC intervals on glutamatergic neurons from CR-formation mice in training days 10 (red symbols; *n* = 11 cells form 6 mice), 17 (blue; *n* = 11 cells from 6 mice) and 18 (green; *n* = 9 cells from 6 mice) as well as those from UPS mice (cyan; *n* = 12 cells from 6 mice). The insert in Figure 3E illustrates that sEPSC intervals at 67% of cumulative probability in these neurons from CR-formation mice are 158 ± 11 ms at day 10 (red bar), 138 ± 14 ms at day 17 (blue) and 174 ± 15 ms at day 18 (green), in comparison with 494 ± 36 ms from UPS mice (cyan; two asterisks, *p* < 0.01). Moreover, Figure 3F shows cumulative probability vs. sEPSC amplitudes on the glutamatergic neurons from CR-formation mice in training days 10 (red symbols), 17 (blue) and 18 (green) as well as those from UPS mice (cyan). The insert in Figure 3F shows that sEPSC amplitudes at 67% of cumulative probability in these neurons from CR-formation mice are 20.0 ± 0.80 pA at day 10 (red bar), 20.60 ± 1.30 pA at day 17 (blue) and 18.30 ± 1.10 pA at day 18 (green), compared with 10.40 ± 0.7 pA from UPS mice (cyan; two asterisks, *p* < 0.01). Therefore, the increase of excitatory synaptic transmission on barrel cortical glutamatergic neurons is maintained regardless of the retrieval inability of cross-modal associative memory.

**Figure 4:**
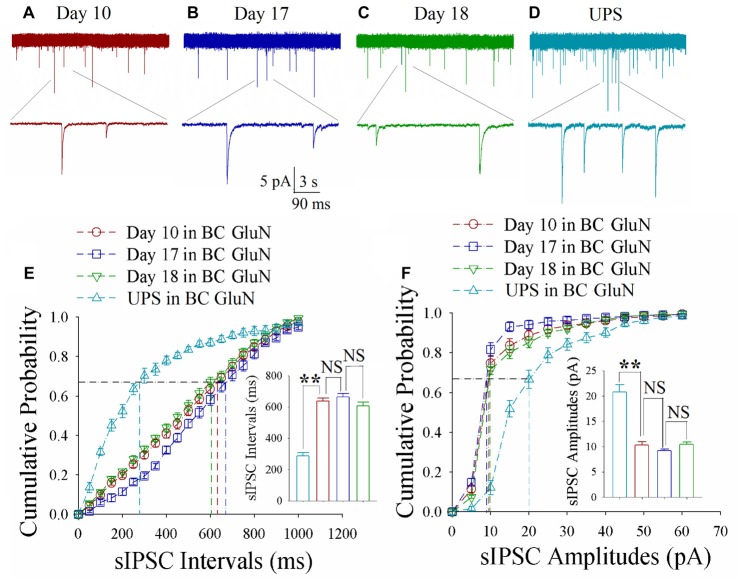
Decreased inhibitory synaptic transmission is maintained at barrel cortical glutamatergic neurons. In UPS mice and CR-formation mice at training days 10, 17 and 18, spontaneous inhibitory postsynaptic currents (sIPSCs) were recorded under voltage-clamp recording on YFP-labeled glutamatergic neurons in brain slices. **(A)** shows sIPSCs at barrel cortical glutamatergic neuron from a CR-formation mouse at training day 10. **(B)** illustrates sIPSCs at barrel cortical glutamatergic neuron from a CR-formation mouse at training day 17. **(C)** shows sIPSCs at barrel cortical glutamatergic neuron from a CR-formation mouse at training day 18. **(D)** shows sIPSCs at barrel cortical glutamatergic neuron from UPS mouse. **(E)** illustrates cumulative probability vs. sIPSC intervals in barrel cortical neurons from UPS mouse (cyan symbols) and from CR-formation mice at training days 10 (red), 17 (blue) and 18 (green). Insert is sIPSC intervals at 67% cumulative probability in the barrel cortical neurons from UPS mouse (cyan bar, 281 ± 19 ms, *n* = 9) and from CR-formation mice at training days 10 (red, 632 ± 22 ms, *n* = 11), 17 (blue, 670 ± 31 ms, *n* = 11) and 18 (green, 604 ± 38 ms, *n* = 9; from left: *p* < 0.001, *p* = 0.418, *p* = 0.107). **(F)** illustrates cumulative probability vs. sIPSC amplitudes in barrel cortical neurons from UPS mouse (cyan symbols) and from CR-formation mice at training days 10 (red), 17 (blue) and 18 (green). Insert is sIPSC amplitude at 67% cumulative probability in the barrel cortical neurons from UPS mouse (cyan bar, 20.8 ± 1.4 pA, *n* = 9) and from CR-formation mice at training days 10 (red, 10.3 ± 0.7 pA, *n* = 11), 17 (blue, 9.2 ± 0.3 pA, *n* = 11) and 18 (green, 10.4 ± 0.5 pA, *n* = 9; from left: *p* < 0.001, *p* = 0.145, *p* = 0.055). A one-way ANOVA with Bonferroni correction for multiple comparisons was performed to test for significant changes, two asterisks represent *p* < 0.01, NS represents no statistical significance.

As shown in Figures 4A–D, spontaneous inhibitory synaptic currents were recorded by whole-cell voltage-clamp on barrel cortical glutamatergic neurons in CR-formation mice and UPS mice. Compared to UPS mice (cyan), sIPSC amplitude and frequency appear decreased in training days 10 (red trace), 17 (blue) and 18 (green). Figure 4E shows cumulative probability vs. sIPSC intervals on the glutamatergic neurons from CR-formation mice in training days 10 (red symbols; *n* = 11 cells from 6 mice), 17 (blue; *n* = 11 cells from 6 mice) and 18 (green; *n* = 9 cells from 6 mice) as well as those from UPS mice (cyan; *n* = 9 cells from 6 mice). The insert in Figure 4E shows that sIPSC intervals at 67% of cumulative probability in these neurons from CR-formation mice are 632 ± 22 ms at day 10 (red bar), 670 ± 31 ms at day 17 (blue) and 604 ± 38 ms at day 18 (green), in comparison with 281 ± 19 ms from UPS mice (cyan; two asterisks, *p* < 0.01). Figure 4F shows cumulative probability vs. sIPSC amplitudes on glutamatergic neurons from CR-formation mice in training days 10 (red symbols), 17 (blue) and 18 (green) as well as those in UPS mice (cyan). The insert in Figure 4F shows that sIPSC amplitudes at 67% of cumulative probability in these neurons from CR-formation mice are 10.3 ± 0.7 pA at day 10 (red bar), 9.2 ± 0.3 pA at day 17 (blue) and 10.4 ± 0.5 pA at day 18 (green), compared to 20.8 ± 1.4 pA from UPS mice (cyan; two asterisks, *p* < 0.01). Therefore, the decrease of inhibitory synaptic transmission on barrel cortical glutamatergic neurons is maintained regardless of the retrieval inability of cross-modal associative memory.

### Excitatory Synapse Upregulation and Inhibitory Synapse Downregulation Change at Motor Cortical Neurons

**Figure 5:**
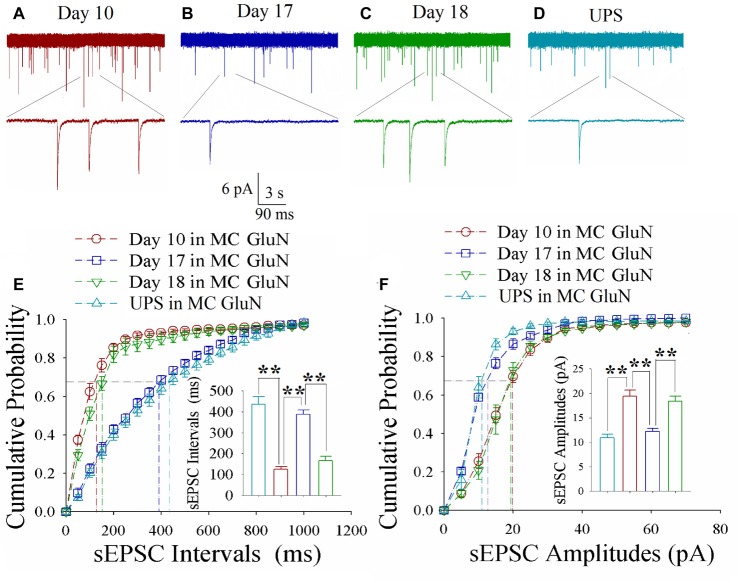
Enhanced excitatory synaptic transmission decays at motor cortical glutamatergic neurons without keeping whisker stimuli (WS)/odor stimuli (OS)-pair. In UPS mice and CR-formation mice at training days 10, 17 and 18, sEPSCs were recorded under the voltage-clamp recording on YFP-labeled glutamatergic neurons in brain slices. **(A)** shows sEPSCs at motor cortical glutamatergic neuron from a CR-formation mouse at training day 10. **(B)** illustrates sEPSCs at the motor cortical glutamatergic neuron from a CR-formation mouse at training day 17. **(C)** illustrates sEPSCs at the motor cortical glutamatergic neuron from a CR-formation mouse at training day 18. **(D)** illustrates sEPSCs at the motor cortical glutamatergic neuron from UPS mouse. **(E)** shows cumulative probability vs. sEPSC intervals in barrel cortical neurons from UPS mouse (cyan symbols) and from CR-formation mice at training days 10 (red), 17 (blue) and 18 (green). Insert shows sEPSC intervals at 67% cumulative probability in the motor cortical neurons from UPS mouse (cyan bar, 437 ± 36 ms, *n* = 12) and from CR-formation mice at training days 10 (red, 125 ± 11 ms, *n* = 11), 17 (blue, 387 ± 22 ms, *n* = 11) and 18 (green, 167 ± 20 ms, *n* = 9; from left: *p* < 0.001, *p* < 0.001, *p* < 0.001). **(F)** illustrates cumulative probability vs. sEPSC amplitudes in the motor cortical neurons from UPS mouse (cyan symbols) and from CR-formation mice at training days 10 (red), 17 (blue) and 18 (green). Insert shows sEPSC amplitude at 67% cumulative probability in the motor cortical neurons from UPS mouse (cyan bar, 10.9 ± 0.8 pA, *n* = 12) and from CR-formation mice at training days 10 (red, 19.4 ± 1.3 pA, *n* = 11), 17 (blue, 12.2 ± 0.6 pA, *n* = 11) and 18 (green, 18.3 ± 1.1 pA, *n* = 9; from left: *p* < 0.001, *p* < 0.001, *p* < 0.001). A one-way ANOVA with Bonferroni correction for multiple comparisons was performed to test for significant changes, two asterisks represent *p* < 0.01, NS represents no statistical significance.

As shown in Figures 5A–D, spontaneous excitatory synaptic currents were recorded by whole-cell voltage-clamp on motor cortical glutamatergic neurons in CR-formation mice in training days 10, 17 and 18 as well as those in UPS mice. Compared to UPS mice (cyan), sEPSC amplitude and frequency appear increased at day 10 (red trace), decreased at day 17 (blue) and increased again at day 18 (green). Figure 5E illustrates cumulative probability vs. sEPSC intervals on glutamatergic neurons from CR-formation mice in training days 10 (red symbols; *n* = 11 cells from 6 mice), 17 (blue; *n* = 11 cells from 6 mice) and 18 (green; *n* = 9 cells from 6 mice) as well as those from UPS mice (cyan; *n* = 12 cells from 6 mice). The insert in Figure 5E shows that sEPSC intervals at 67% of cumulative probability in these neurons from CR-formation mice are 125 ± 11 ms at day 10 (red bar), 387 ± 22 ms at day 17 (blue) and 167 ± 20 ms at day 18 (green), compared to 437 ± 36 ms from UPS mice (cyan; two asterisks, *p* < 0.01). Moreover, Figure 5F illustrates cumulative probability vs. sEPSC amplitudes on glutamatergic neurons from CR-formation mice in training days 10 (red symbols), 17 (blue) and 18 (green) as well as those from UPS mice (cyan). The insert in Figure 5F illustrates that sEPSC amplitudes at 67% of cumulative probability in these neurons from CR-formation mice are 19.4 ± 1.3 pA at day 10 (red bar), 12.2 ± 0.6 pA at day 17 (blue) and 18.3 ± 1.1 pA at day 18 (green), compared with 10.9 ± 0.8 pA from UPS mice (cyan; two asterisks, *p* < 0.01). Therefore, the increase of excitatory synaptic transmission on the motor cortical glutamatergic neurons is decayed in the retrieval inability of cross-modal associative memory.

**Figure 6:**
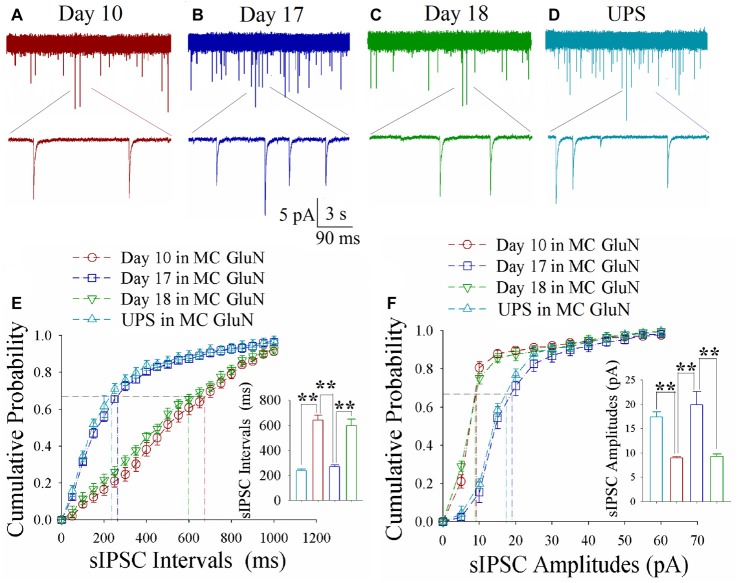
Decreased inhibitory synaptic transmission decays at motor cortical glutamatergic neurons without keeping WS/OS-pair. In UPS mice and CR-formation mice at training days 10, 17 and 18, sIPSCs were recorded under the voltage-clamp recording on YFP-labeled glutamatergic neurons in brain slices. **(A)** shows sIPSCs at motor cortical glutamatergic neuron from a CR-formation mouse at training day 10. **(B)** illustrates sIPSCs at the motor cortical glutamatergic neuron from a CR-formation mouse at training day 17. **(C)** illustrates sIPSCs at the motor cortical glutamatergic neuron from a CR-formation mouse at training day 18. **(D)** illustrates sIPSCs at the motor cortical glutamatergic neuron from UPS mouse. **(E)** shows cumulative probability vs. sIPSC intervals in barrel cortical neurons from UPS mouse (cyan symbols) and from CR-formation mice at training days 10 (red), 17 (blue) and 18 (green). Insert shows sIPSC intervals at 67% cumulative probability in the motor cortical neurons from UPS mouse (cyan bar, 235 ± 16 ms, *n* = 9) and from CR-formation mice at training days 10 (red, 641 ± 37 ms, *n* = 11), 17 (blue270 ± 14 ms, *n* = 11) and 18 (green, 595 ± 53 ms, *n* = 9; from left: *p* < 0.001, *p* < 0.001, *p* < 0.001). **(F)** illustrates cumulative probability vs. sIPSC amplitudes in the motor cortical neurons from UPS mouse (cyan symbols) and from CR-formation mice at training days 10 (red), 17 (blue) and 18 (green). Insert shows sIPSC amplitude at 67% cumulative probability in the motor cortical neurons from UPS mouse (cyan bar, 17.4 ± 1 pA, *n* = 9) and from CR-formation mice at training days 10 (red, 9 ± 0.2 pA, *n* = 11), 17 (blue, 20 ± 2.6 pA, *n* = 11) and 18 (green, 9.2 ± 0.5 pA, *n* = 9; from left: *p* < 0.001, *p* = 0.002, *p* = 0.002). A one-way ANOVA with Bonferroni correction for multiple comparisons was performed to test for significant changes, two asterisks represent *p* < 0.01, NS represents no statistical significance.

As shown in Figures 6A–D, spontaneous inhibitory synaptic currents were recorded by whole-cell voltage-clamp on motor cortical glutamatergic neurons in CR-formation mice and UPS mice. Compared to UPS mice (cyan), sIPSC amplitude and frequency appear decreased in training day 10 (red trace), returned to baseline at day 17 (blue) and decreased again ay day 18 (green). Figure 6E shows cumulative probability vs. sIPSC intervals on glutamatergic neurons from CR-formation mice in training days 10 (red symbols; *n* = 11 cells from 6 mice), 17 (blue; *n* = 11 cells from 6 mice) and 18 (green; *n* = 9 cells from 6 mice) as well as those from UPS mice (cyan; *n* = 9 cells from 6 mice). The insert in Figure 6E illustrates that sIPSC intervals at 67% of cumulative probability in these neurons from CR-formation mice are 641 ± 37 ms at day 10 (red bar), 270 ± 14 ms at day 17 (blue) and 595 ± 53 ms at day 18 (green), in comparison with 235 ± 16 ms from UPS mice (cyan; two asterisks, *p* < 0.01). Figure 6F shows cumulative probability vs. sIPSC amplitudes on the glutamatergic neurons from CR-formation mice in training days 10 (red symbols), 17 (blue) and 18 (green) as well as those from UPS mice (cyan). The insert in Figure 6F shows that sIPSC amplitudes at 67% of cumulative probability in these neurons from CR-formation mice are 9 ± 0.2 pA at day 10 (red bar), 20 ± 2.6 pA at day 17 (blue) and 9.2 ± 0.5 pA at day 18 (green), compared with 17.4 ± 1 pA from UPS mice (cyan; two asterisks, *p* < 0.01). Therefore, the decrease of inhibitory synaptic transmission on the motor cortical glutamatergic neurons is returned to the baseline in the retrieval inability of cross-modal associative memory.

### Decayed Neural Plasticity in the Motor Cortex Is Closely Correlated to the Extinction of Associative Memory

**Figure 7:**
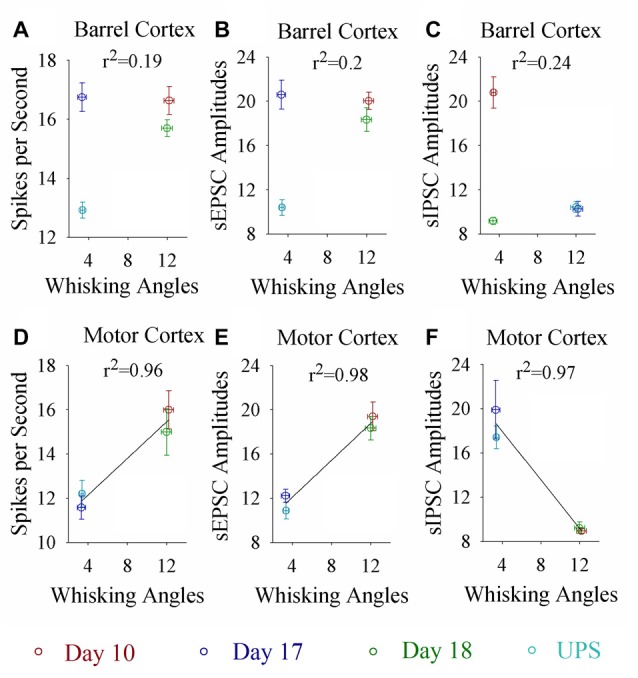
Whisking angles in response to the odor-test at CR-formation mice are correlated with synaptic strength and spike ability in the motor cortex, but not in the barrel cortex, during associative memory formation, extinction and reestablishment. The strengths of associative memory, such as the angles of whisker fluctuation in response to the odor-test, in CR-formation mice at training days 10 (red symbols), 17 (blue) and 18 (green) as well as UPS mice (cyan) are plotted in *X*-axis. The amplitudes of sEPSCs and sIPSCs at 67% cumulative probability as well as the number of spikes induced by 3.0 normalized stimuli in the input-output curves are plotted in *Y*-axis. **(A)** shows spikes per second vs. whisking angles in the barrel cortex (linear regression, *r*^2^ = 0.19, *p* = 0.569). **(B)** shows sEPSC amplitudes vs. whisking angles in the barrel cortex (linear regression, *r*^2^ = 0.2, *p* = 0.550). **(C)** shows sIPSC amplitudes vs. whisking angles in the barrel cortex (linear regression, *r*^2^ = 0.24, *p* = 0.512). **(D)** shows spikes per second vs. whisking angles in the motor cortex (linear regression, *r*^2^ = 0.96, *p* = 0.022). **(E)** shows sEPSC amplitudes vs. whisking angles in the motor cortex (linear regression, *r*^2^ = 0.98, *p* = 0.012). **(F)** shows sIPSC amplitudes vs. whisking angles in the motor cortex (linear regression, *r*^2^ = 0.97, *p* = 0.016).

If the dynamical changes of neural plasticity in either barrel cortex or motor cortex are correlated with the dynamical change of cross-modal associative memory, the establishment and extinction of associative memory are set by neuronal plasticity. To test this possibility, we take the following parameters into our analysis. The strengths of associative memory, such as the angles of whisker fluctuation in response to the odor-test, in CR-formation mice in training days 10, 17 and 18 as well as UPS mice are plotted in *X*-axis. The amplitudes of sEPSCs and sIPSCs at 67% cumulative probability as well as the number of spikes induced by 3.0 normalized stimuli in input-output curves are plotted in *Y*-axis. As demonstrated in Figure 7, the changes of associative memory strength are linearly correlated to synaptic activity strength and spiking ability in the motor cortices, but not in the barrel cortices. Thus, cellular mechanisms underlying the dynamical change of associative memory, especially extinction, are correlated to neural plasticity at the motor cortices in terms of the increases of excitatory synaptic transmission and spike ability as well as the decrease of inhibitory synaptic transmission on the glutamatergic neurons.

## Discussion

**Figure 8:**
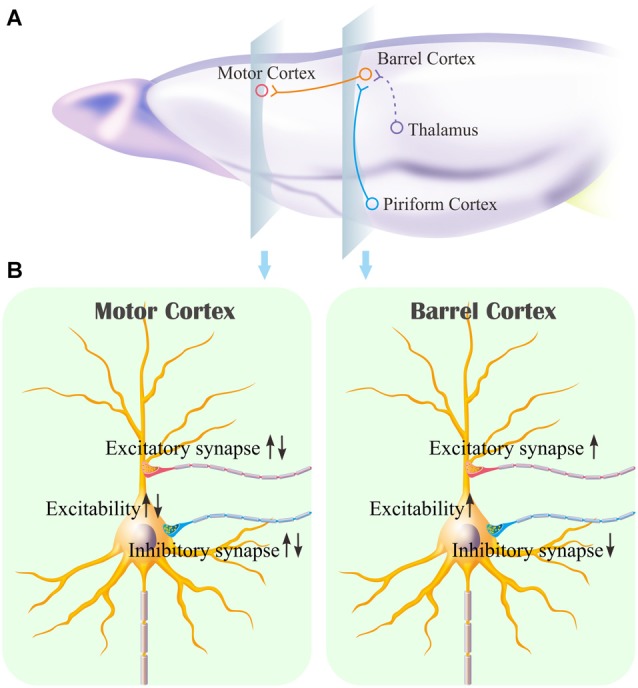
Associative learning induces the synapse innervations from the piriform cortex to the barrel cortex where associative memory cells are recruited as well as the neuronal plasticity in the barrel cortex and the motor cortex. **(A)** Associative memory cells in the barrel cortex (red) receive the synapse innervations newly from the piriform cortex (blue) and innately from the thalamus (green). These associative memory cells project their axons toward the motor cortex. **(B)** illustrates persistent plasticity on a glutamatergic neuron in the barrel cortex, and dynamic plasticity on a glutamatergic neuron in the motor cortex based on use-dependence.

The mice that receive paired whisker and olfaction stimulations exhibit odorant-induced whisker motion, in which the odor signal induces whisker signal recall (cross-modal associative memory) and subsequent whisker motion (Wang et al., 2015; Gao et al., 2016; Yan et al., 2016). The cross-modal associative memory appears decayed without paired stimulation for 1 week and recovered by paired stimulations for an additional day (Figure 1). This model presents memory formation, decay and recovery. In terms of their cellular mechanisms, our study shows that the increases of excitatory synaptic transmission and spiking ability as well as the decrease of inhibitory synaptic transmission on glutamatergic neurons are maintained in the barrel cortices, whereas the dynamic changes of neural plasticity in the motor cortex are correlated to the establishment, decay and recovery of associative memory (Figures 2–7; Supplementary Figures S1, S2). These results indicate the maintenance of associative memory in sensory cortices and the retrieval inability of associative memory in behavior-related cortices (Figure 8).

Our studies show that odorant-induced whisker motion and whisker-induced olfaction response (examples of cross-modal associative memories) are based on mutual synaptic innervation between barrel and piriform cortices, associative memory cells in these sensory cortices and their coordinated plasticity (Wang et al., 2014, 2015, 2017; Gao et al., 2016; Wang J.-H. et al., 2016; Yan et al., 2016). These recruited mutual synaptic innervations on associative memory cells in the sensory cortices may not be faded in their structures, such that their upregulated functions to store associated signals are maintained well (Figures 2–4). On the other hand, the behavior-related cortices respond to conditioned signals (Naya et al., 2003; Takehara-Nishiuchi and McNaughton, 2008; Viskontas, 2008; Cai et al., 2016) from associative memory cells in the sensory cortices (Wang and Cui, 2017). The plasticity of activity strengths at these secondary associative memory cells in behavior-related cortices depends on the activity of primary associative memory cells in the sensory cortices, i.e., activity-dependent plasticity (Wang et al., 2017). Without paired stimulations, the structures of mutual synapse innervation and primary associative memory cells in the sensory cortices can be maintained, however, neuronal plasticity in the behavior-related cortices may be decayed. This is a “false” memory extinction, i.e., memory retrieval inability, since it can be recovered by the paired training for an additional day (Figures 1–6).

In many studies of cellular mechanisms underlying memory formation, neural plasticity has been found to occur during associative memory, in which excitatory synaptic function and neuronal spikes are upregulated in the brain (Honey and Good, 2000; Blair et al., 2001; Christian and Thompson, 2003; Jones et al., 2003; Silva, 2003; Zhang et al., 2004; Dityatev and Bolshakov, 2005; Fanselow and Poulos, 2005; Weeks et al., 2007; Frey and Frey, 2008; Nikitin et al., 2008; Rosselet et al., 2011) and inhibitory synaptic function is downregulated in the sensory cortices (Gao et al., 2016; Yan et al., 2016). Less studies have been performed that compare neuronal plasticity between the sensory and motor cortices, neuronal plasticity dynamics during memory formation, extinction and recovery, as well as coordinated plasticity among excitatory and inhibitory synapses. Our studies in these combinations bring the insight into potential mechanisms for information storage and memory extinction.

In real life, the recall of stored signals can be spontaneous or cue-evoked. The storage of some signals with impressive memory, which leads to extreme happiness, fear and addiction, may be due to the possibility that a large amount of associative memory cells is recruited (Wang and Cui, 2017; Wang et al., 2017). The spontaneous activation of such associative memory cells makes the associated signals to be recalled intrinsically, leading to associative thinking and logical reasoning. On the other hand, some signals are retrieved from the sensory cortices by sensory cues similar to or equal to these signals. Their evoked recalls may be due to less recruitment of associative memory cells and/or the decay of neuronal plasticity in behavior-related cortices for memory presentation. In this regard, mutual synaptic innervation and associative memory cells may determine the specificity of the stored signals for long-time maintenance, whereas neuronal plasticity, especially in behavior-related cortices, influences the presentation of memorized signals. Based on neural circuits from primary associative memory cells in sensory cortices (Wang et al., 2014, 2015, 2017; Gao et al., 2016; Wang J.-H. et al., 2016; Yan et al., 2016) to secondary associative memory cells in behavior- cognition- and emotion-relevant brain regions (Naya et al., 2003; Takehara-Nishiuchi and McNaughton, 2008; Viskontas, 2008; Cai et al., 2016), activating any of these regions induces memory presentation (Ehrlich et al., 2009; Pape and Pare, 2010; Liu et al., 2012; Li et al., 2013; Xu and Südhof, 2013; Otis et al., 2017; Yokose et al., 2017).

It remains to be investigated why neuronal plasticity at associative memory cells in sensory cortices can be maintained well, whereas neuronal plasticity in behavior-related cortices undergoes a decay without paired-stimulation. Neural plasticity at associative memory cells in sensory cortices is based on new synaptic innervations among these areas. These newly formed synapses with complete structure and function (Wang et al., 2015; Gao et al., 2016) may not be ruined easily, so that the associative memory cells, neuronal plasticity and associative memory are well maintained. On the other hand, innate synaptic innervations from sensory cortices to behavior-related cortices show functional plasticity based on the activities of sensory cortices (Figures 2–6), which is use-dependent and disappears without the paired stimulations. In terms of reestablishment of neuronal and synaptic plasticity, we assume that the decay of neuronal plasticity is re-boosted at silent synapses and/or the neurons are reactivated in the motor cortices. The detailed molecular mechanisms in both sensory and motor cortices remain to be addressed.

Current reports indicate that associative memory cells and their plasticity play important roles in this cross-modal associative memory (Wang et al., 2015; Gao et al., 2016; Yan et al., 2016), in which the new synaptic innervation from the co-activated cortices is required (Wang et al., 2015, 2017; Gao et al., 2016; Wang J.-H. et al., 2016). As epigenetic processes are presumably involved in memory (Molfese, 2011; Kaas et al., 2013; Landry et al., 2013; Lattal and Wood, 2013; Woldemichael et al., 2014; Yan et al., 2016), we are studying how these molecules, such as miRNA-324/miRNA-133a and their downstream targets, influence new synaptic innervations, recruit associative memory cells and induce plasticity in both sensory and motor cortices for memory establishment, extinction and reestablishment.

## Author Contributions

RGu, RGe, SZ, YL, XZ, LH, SG, WL, SC and SW contributed to experiments and data analyses. J-HW contributed to project design and article writing. RGu, RGe and SZ equally contributed to this study.

## Conflict of Interest Statement

The authors declare that the research was conducted in the absence of any commercial or financial relationships that could be construed as a potential conflict of interest.
