# Supplementary material for: Associative Memory Extinction Is Accompanied by Decayed Plasticity at Motor Cortical Neurons and Persistent Plasticity at Sensory Cortical Neurons
Source: Front Cell Neurosci. 2017 Jun 14;11:168. doi: 10.3389/fncel.2017.00168 (PMC5469894; doi:10.3389/fncel.2017.00168)

Submission to: Frontiers in Cellular Neuroscience

Associative memory extinction is accompanied by decayed plasticity at motor cortical neurons and persistent plasticity at sensory cortical neurons

Rui Guo[1#], Rongjing Ge[1#], Shidi Zhao[1#], Yulong Liu[1], Xin Zhao[1], Li Huang[1], Sodong Guan[1], Wei Lu[3], Shan Cui[2], Shirlene Wang[4], and Jin-Hui Wang[1,2,3]*

1) Department of Pathophysiology, Bengbu Medical College, Anhui 233000
2) Institute of Biophysics and University of Chinese Academy of Sciences, Beijing China 100101
3) Qingdao University, School of Pharmacy, Qingdao Shandong China 266021
4)Department of Psychiatry and Behavioral Sciences, Northwestern University, Feinberg School of Medicine, Chicago IL 60091, USA

Running title: Cell-specific mechanism for memory

Key words: learning, memory, glutamate, GABA, neuron, synapse, barrel cortex and homeostasis

Word counts: abstract, 227; text, 7367

Corresponding author:

Jin-Hui Wang, Ph.D. & MD
Brain and Cognitive Sciences
The Institute of Biophysics, Chinese Academy of Sciences
15 Datun Road, Beijing China 100101
jhw@sun5.ibp.ac.cn; 86-10-64888472

Figure Legends

Figure S1 Whisker retraction duration in response to the odor-test at CR-formation mice are correlated with synaptic strength and spike ability in the motor cortex, but not in the barrel cortex, during associative memory formation, extinction and reestablishment. The strengths of associative memory, such as whisker retraction duration in response to the odor-test, in CR-formation mice at training days 10 (red symbols), 17 (blue) and 18 (green) as well as UPS mice (cyan) are plotted in X-axis. The amplitudes of sEPSCs and sIPSCs at 67% cumulative probability as well as the number of spikes induced by 3.0 normalized stimuli in the input-output curves are plotted in Y-axis. **A)** shows spikes per second versus whisker retraction duration in the barrel cortex. **B)** shows sEPSC amplitudes versus whisker retraction duration in the barrel cortex. **C)** shows sIPSC amplitudes versus whisker retraction duration in the barrel cortex. **D)** shows spikes per second versus whisker retraction duration in the motor cortex. **E)** shows sEPSC amplitudes versus whisker retraction duration in the motor cortex. **F)** shows sIPSC amplitudes versus whisker retraction duration in the motor cortex.

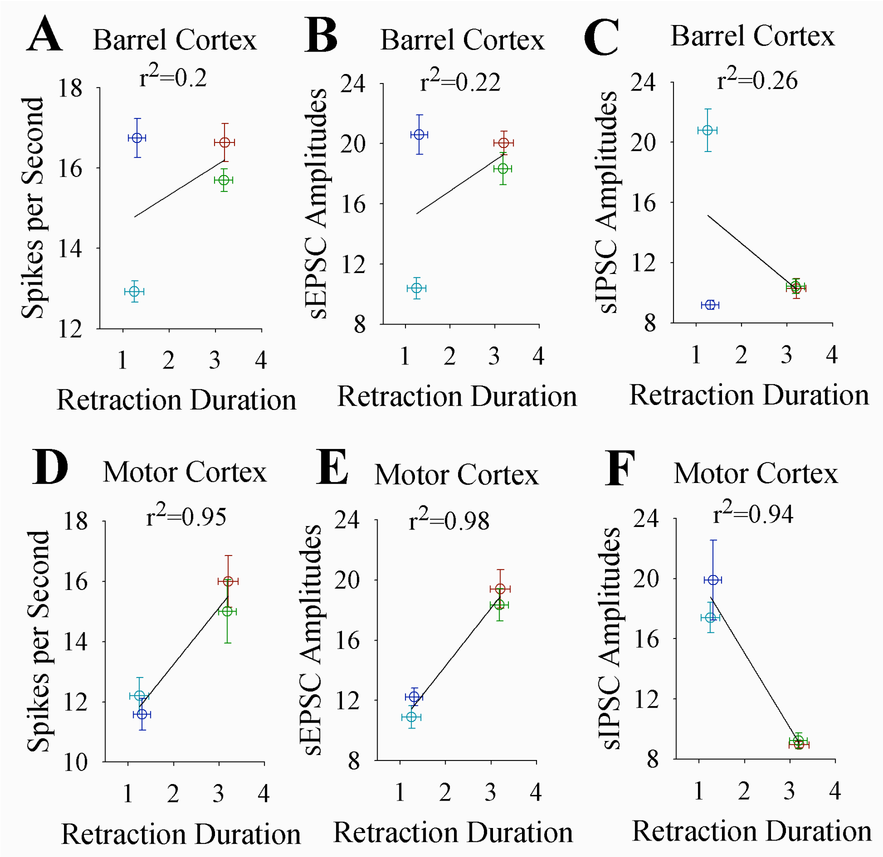

Figure S2 Whisking frequency in response to the odor-test at CR-formation mice are correlated with synaptic strength and spike ability in the motor cortex, but not in the barrel cortex, during associative memory formation, extinction and reestablishment. The strengths of associative memory, such as whisking frequency in response to the odor-test, in CR-formation mice at training days 10 (red symbols), 17 (blue) and 18 (green) as well as UPS mice (cyan) are plotted in X-axis. The amplitudes of sEPSCs and sIPSCs at 67% cumulative probability as well as the number of spikes induced by 3.0 normalized stimuli in the input-output curves are plotted in Y-axis. **A)** shows spikes per second versus whisking frequency in the barrel cortex. **B)** shows sEPSC amplitudes versus whisking frequency in the barrel cortex. **C)** shows sIPSC amplitudes versus whisking frequency in the barrel cortex. **D)** shows spikes per second versus whisking frequency in the motor cortex. **E)** shows sEPSC amplitudes versus whisking frequency in the motor cortex. **F)** shows sIPSC amplitudes versus whisking frequency in the motor cortex.

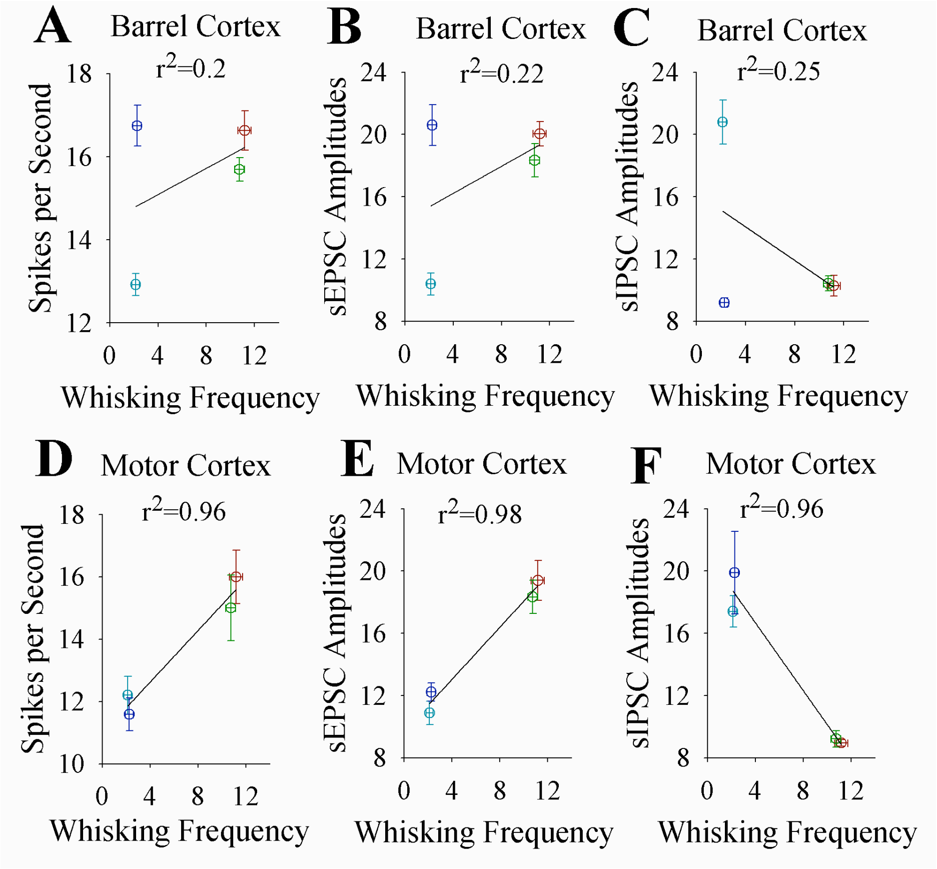

Supplement: Supplementary file 1 [file Presentation_1.pdf]
